# Highly Sensitive Si-Based Electrolyte-Gated Transistor Array for Multiplexed Detection of Arboviruses

**DOI:** 10.3390/mi16111279

**Published:** 2025-11-13

**Authors:** Seonghwan Shin, Jeonghyeon Do, Jongmin Son, Jeong-Soo Lee

**Affiliations:** 1 Department of Electrical Engineering, Pohang University of Science and Technology (POSTECH), Pohang 37673, Republic of Korea; ssh3290a@postech.ac.kr (S.S.); toaru124@postech.ac.kr (J.D.); jmson@postech.ac.kr (J.S.); 2 Innovative General Electronic Sensor Technology (i-GEST) Co., Ltd., Pohang 37673, Republic of Korea

**Keywords:** electrolyte-gated transistor, multiplexed biosensor, aptamer functionalization, arbovirus detection, point-of-care diagnostics

## Abstract

Multiplexed detection of arboviruses using a 4 × 4 Si-based electrolyte-gated transistor (EGT) array functionalized with specific aptamers has been investigated. The Si-based EGTs were fabricated using conventional Si microfabrication processes. The EGTs showed excellent intrinsic electrical characteristics, including a low threshold voltage of 0.8 V, a sub-threshold swing of 75 mV/dec, and a gate leakage of <10 pA, ensuring uniform device performance with low device-to-device variation. Aptamers specific to the yellow fever virus nonstructural protein 1 (YF), dengue virus nonstructural protein 1 (DN), and chikungunya virus envelope protein 2 (CHK) were functionalized on EGT arrays to evaluate individual and multiplexed detection. In individual-target detections, concentration-dependent negative shifts in threshold voltage were observed, and relevant limits of detection (LOD) as low as 38.6 pg/mL, 95.2 pg/mL, and 1.6 ng/mL were extracted for YF, DN, and CHK, respectively. In multiplexed detections, sensitivities decreased and variations increased relative to the individual responses, resulting in higher LODs. The extracted LODs were 0.2 ng/mL, 0.6 ng/mL, and 2.8 ng/mL for YF, DN, and CHK, respectively, which are lower than those reported for other methods. These results suggest that Si-based EGT arrays are promising as a scalable, low-cost, and highly sensitive biosensing platform for multiplexed arbovirus detection and point-of-care diagnostics.

## 1. Introduction

Arboviruses, including yellow fever virus, dengue virus, and chikungunya virus, remain significant global health concerns, particularly in tropical and subtropical regions [1,2,3]. These viruses often co-circulate and can even co-infect individuals, causing overlapping flu-like symptoms such as fever, headache, and muscle pain [4,5]. Such clinical similarities make differential diagnosis challenging in endemic areas, frequently leading to misdiagnosis and delayed treatment [6,7]. Conventional methods, such as polymerase chain reaction (PCR) and enzyme-linked immunosorbent assays (ELISA), offer high accuracy but require expensive equipment, skilled personnel, and long processing times [8,9]. For point-of-care applications, there is an urgent need for rapid, low-cost, and portable diagnostic tools capable of multiplex detection of these viruses.

Multiplexed biosensing has been explored using various platforms, including optical, acoustic, colorimetric, and magnetic bead-based approaches [10,11,12,13,14,15,16]. While these methods enable simultaneous detection, their reliance on bulky equipment or complex assay procedures limits their applicability for point-of-care settings. Field-effect transistor (FET) biosensors, on the other hand, offer high sensitivity, rapid and label-free responses, and are compatible with miniaturization and low-cost fabrication. Multiplexing has been demonstrated in various FET architectures, including hydrogel-gated graphene transistors, dual-gate oxide thin-film transistors, and silicon nanowire FETs [17,18,19]. However, these BioFETs often face challenges such as a limited sensing area or additional parasitic components when connecting the external gate structure [20,21].

Recently, EGTs have emerged as a promising platform for biosensing [22,23]. The use of relatively larger gate electrodes could increase receptor density and enhance the probability of receptor-target binding events [24,25,26]. Furthermore, Si-based EGTs are advantageous over other organic/2D material devices due to their environmental stability, low power consumption, and compatibility with microfabrication processes [27,28,29].

Here, the multiplexed detection of arboviruses using an aptamer-functionalized EGT array is demonstrated. A 4 × 4 Si-based EGT array enabling multiplexed detections was fabricated using a top-down CMOS-compatible process. The electrical characteristics across all 16 devices were sufficiently reproducible to ensure a reliable baseline for multiplexed detection. DNA aptamers specific to YF, DN, and CHK were employed as biorecognition elements. Both individual and multiplexed detections were characterized and evaluated in terms of sensitivity, LOD, and selectivity.

## 2. Materials and Methods

### 2.1. Material Preparation

Phosphate-buffered saline (PBS, pH 7.4) was purchased from Sigma-Aldrich (Burlington, VT, USA). Thiol-modified aptamers specific to YF, DN, and CHK were obtained from Bionics (Daejeon, Republic of Korea). YF, West Nile Virus nonstructural protein 1 (WN), and Zika Virus nonstructural protein 1 (ZIK) were purchased from Fitzgerald (Acton, MA, USA). DN and CHK were purchased from Sino Biological (Beijin, China).

### 2.2. Fabrication of EGTs

**Figure 1:**
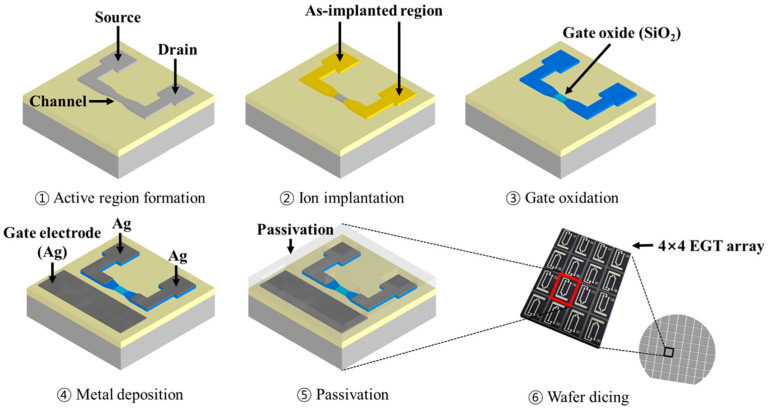
A schematic of the fabrication process of the EGT array.

EGT arrays were fabricated using a top-down CMOS-compatible process on an 8-inch p-type silicon-on-insulator (SOI) wafer (10 Ω·cm, (100)). Figure 1 shows a schematic of the EGT fabrication steps. The SOI wafer comprised a 100 nm Si layer and a 400 nm buried oxide. Lithographic patterning of the active regions (source, drain, and channel) using a KrF scanner was performed, followed by inductively coupled plasma reactive-ion etching (ICP-RIE). Arsenic ions at a dose of 5 × 10^15^ cm^−2^ were implanted into the source and drain and then activated by rapid thermal annealing (RTA, 1000 °C, 20 s). A thin SiO_2_ gate dielectric (5 nm) was subsequently grown by thermal oxidation. Then, metal electrodes for the gate, source, and drain interconnects were defined by I-line stepper lithography, followed by a lift-off and the deposition of Ti/Ag (50 nm/500 nm) using e-beam evaporation. Finally, a 3 µm SU-8 layer was patterned as a passivation layer, except for the gate, channel, and contact pads. To enable multiplexing capability, the wafer was diced into multiple dies, each containing 4 × 4 EGTs (16 devices per die).

### 2.3. Aptamer Functionalization and Detection Protocol

**Figure 2:**
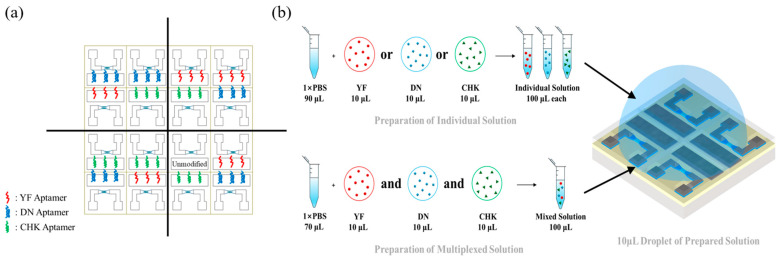
(**a**) 4 × 4 EGT array with aptamer-functionalized regions. The responses of each target are characterized using five different EGTs in the EGT array. The unmodified EGT in the 4th quadrant is used for the selectivity tests. (**b**) Detection scheme using individual-target and multiplexed droplets.

Aptamers functionalized with thiol groups at their termini were exposed to the Ag electrode of the device to bind [30,31]. Before aptamer functionalization, the thiol-ended aptamers (in 1 × PBS) were activated by heating at 95 °C for 5 min, then cooling at 4 °C for 30 min. Then, 1 µL of the aptamer solution was dropped onto each EGT gate surface. After one hour, the devices were rinsed with 1 × PBS and deionized water, and then gently dried with N_2_ gas. Aptamers specific to YF, DN, and CHK were functionalized across 4 × 4 EGT arrays. Figure 2a shows a representative functionalization of the array, in which devices were coated with three different aptamers to minimize variability during characterization. For individual-target testing, a 10 µL aliquot of a solution containing only one viral protein was added to 90 µL of 1 × PBS. A 10 µL droplet of this solution was then applied to each quadrant of the array and incubated for 30 min. For multiplexed detection, 10 µL of each of the three viral proteins was added with 70 µL of 1 × PBS, and 10 µL of this multiplexed solution was similarly applied to each quadrant and incubated for 30 min, as shown in Figure 2b.

### 2.4. Electrical Characterization

Electrical measurements were carried out using a semiconductor parameter analyzer (Keithley 4200, Keithley, Solon, OH, USA). The drain current (*I_D_*) was measured at a constant drain voltage (*V_D_*) of 0.1 V with the source grounded (*V_S_* = 0 V). The gate voltage (*V_G_*) was applied through the electrolyte (0.01 × PBS buffer solution) and swept from 0 to 1 V with a step size of 0.05 V. The *I_D_* was limited to 10^−7^ A to prevent device degradation during characterization.

## 3. Results and Discussion

### 3.1. Intrinsic Electrical Characteristics

**Figure 3:**
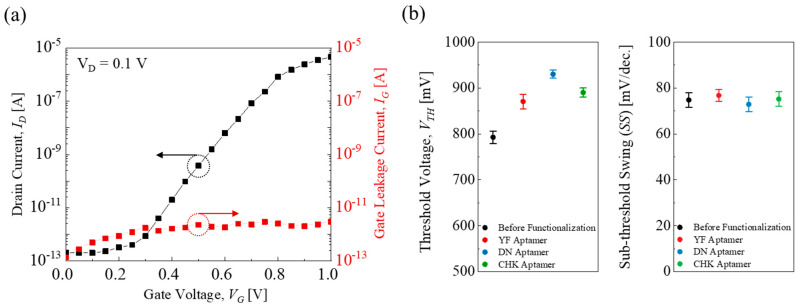
(**a**) A representative transfer characteristic (*I_D_V_G_*) and gate leakage current (*I_G_*) in an EGT device. (**b**) *V_TH_* and *SS* variation before and after YF-, DN-, and CHK-aptamer functionalization.

Figure 3a shows a representative transfer curve (*I_D_*–*V_G_*) with a low gate leakage current (*I_G_*) of less than 10 pA. Figure 3b shows the threshold voltage (*V_TH_*) and sub-threshold swing (*SS* ≡ d*V_G_*/dlog (*I_D_*)) in the EGT array. After aptamer functionalization, the transfer curves *I_D_* showed a right shift, indicating a positive shift in threshold voltage due to the formation of a dipole layer induced by the negatively charged aptamer molecules [25,32]. The average *V_TH_* values were 792 ± 14 mV, 870 ± 16 mV, 930 ± 9 mV, and 890 ± 10 mV for before, YF-, DN-, and CHK-aptamer functionalization, respectively, as determined from the *gm* max method [33]. In contrast, the *SS* values remained nearly unchanged regardless of the surface modification, with an average of 75 ± 3 mV/dec. These results indicate that the EGT array could provide a solid baseline for subsequent multiplexed-sensing characterizations.

### 3.2. Individual-Target Responses

**Figure 4:**
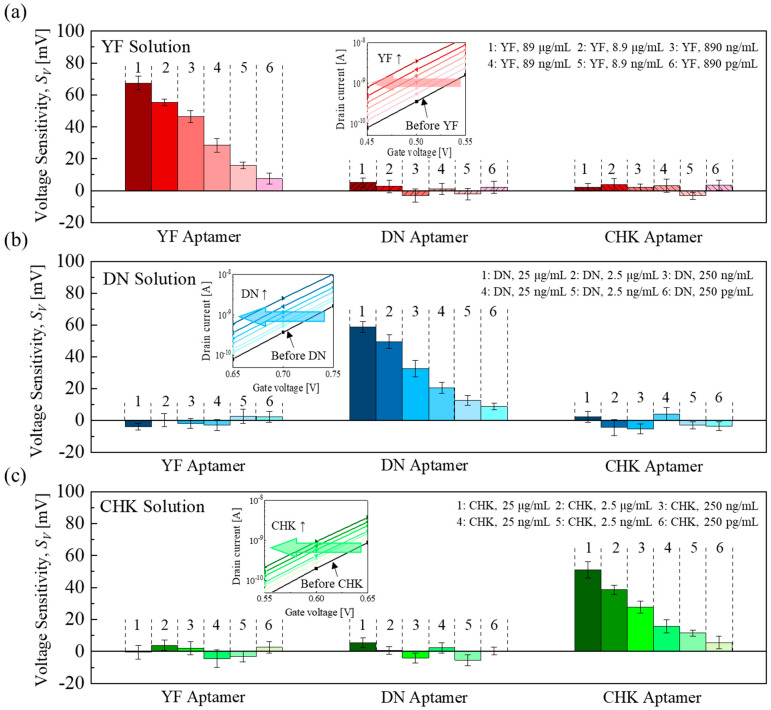
Individual-target detection: *S_V_* responses of YF−, DN−, and CHK−aptamer functionalized EGTs to (**a**) the YF solution, (**b**) the DN solution, and (**c**) the CHK solution. Error bars indicate the standard deviation (±1σ) derived from measurements of five EGTs (n = 5) across the array. Inset: representative transfer curve.

The voltage sensitivity (*S_V_*) was defined as the gate voltage shift (*S_V_* = *V_G_Aptamer_* − *V_G_Target_*), where *V_G_Aptamer_* and *V_G_Target_* are the gate voltages before and after target solution exposure, respectively, measured at a drain current of 1 nA. Figure 4a–c shows the *S_V_* of the aptamer-functionalized EGTs when exposed to the corresponding target solutions (YF: 890 pg/mL–89 μg/mL; DN and CHK: 250 pg/mL–25 μg/mL). Those aptamer–target pairs showed distinct, concentration-dependent responses, while the non-matching pairs showed negligible responses, indicating the high selectivity of the aptamer-functionalized EGT array toward each target analyte.

All the electrical responses show an increase in S_V_ as the target concentration increases (or a lateral decrease in V_TH_ in transfer curves). This negative shift arises from the formation of a dipole layer at the gate surface with the aptamer-target conjugate [25,28,34,35]. The dipole generates a positive effective dipole potential directed toward the gate electrode, which increases the surface potential of the channel. To maintain the same channel potential for a given drain current, this dipole-induced potential must be compensated by lowering the applied gate voltage, resulting in a reduced flat-band voltage (V_FB_). Consequently, the transfer curve shifts toward lower gate voltages, appearing as a negative V_TH_ shift.

**Figure 5:**
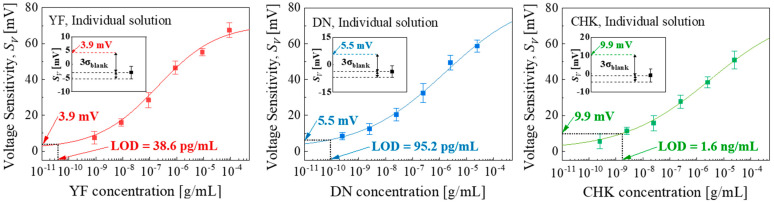
Individual-target detection: Logistic fits of *S_V_* versus target concentration. Error bars indicate the standard deviation (±1σ) derived from measurements of five EGTs (n = 5) across the array. Inset: *S_V_* of blank sample (1 × PBS without any target) at the LOD using the three-sigma method.

Figure 5 shows the *S_V_* and LOD as a function of concentration for each individual-target detection. The experimental data was well fitted with logistic calibration curves, which were subsequently used to calculate the LODs. The fitted equations were *S_V_* = 71.8 × [YF]^0.36^/(3.8 × 10^−3^ + [YF]^0.36^), *S_V_* = 96.5 × [DN]^0.25^/(4.3 × 10^−2^ + [DN]^0.25^), and *S_V_* = 80.6 × [CHK]^0.26^/(3.8 × 10^−2^ + [CHK]^0.26^), respectively. The corresponding blank replicates showed *S_V_* of −3.0 ± 2.3 mV (YF), −3.8 ± 3.1 mV (DN), and −0.9 ± 3.6 mV (CHK), yielding LODs of 38.6 pg/mL for YF, 95.2 pg/mL for DN, and 1.6 ng/mL for CHK, respectively. The blank sample (no target) showed a negligible response, confirming that the observed responses originated from the specific target binding, as shown in the insets of Figure 5.

### 3.3. Multiplexed-Targets Responses

**Figure 6:**
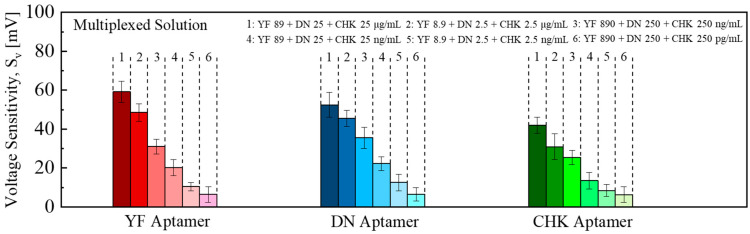
Multiplex detection: *S_V_* vs. target concentration for YF, DN, and CHK aptamer-functionalized EGTs. Error bars indicate the standard deviation (±1σ) derived from measurements of five EGTs (n = 5) across the array.

To further evaluate the sensing performance under more practical conditions, multiplexed detection was conducted by exposing mixtures of the three target analytes to the aptamer-functionalized EGT array. Figure 6 shows the *S_V_* for YF-, DN-, and CHK-functionalized EGTs when exposed to multiplexed solutions. The *I_D_*–*V_G_* curves shifted toward negative V_G_ values with concentration-dependent shifts, similar to those observed in individual detection.

**Figure 7:**
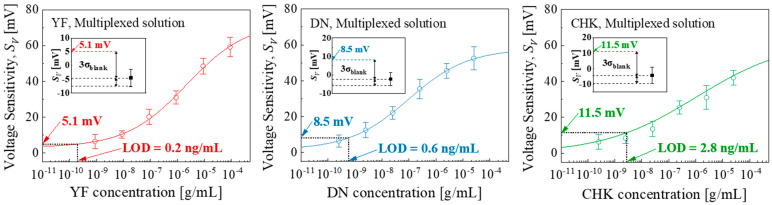
Multiplexed detection: Logistic fits *S_V_* versus target concentration. Error bars indicate the standard deviation (±1σ) derived from measurements of five EGTs (n = 5) across the array. Inset: *S_V_* of blank sample (1 × PBS without any target) at the LOD using the three-sigma method.

Figure 7 shows the *S_V_* variation with different target solutions obtained in the multiplexed detection. The fitted equations were *S_V_* = 78.2 × [YF]^0.33^/(1.4 × 10^−2^ + [YF]^0.33^), *S_V_* = 59.0 × [DN]^0.37^/(2.5 × 10^−3^ + [DN]^0.37^), and *S_V_* = 63.0 × [CHK]^0.27^/(2.5 × 10^−2^ + [CHK]^0.27^). The corresponding blank replicates showed *S_V_* and LOD of −4.5 ± 3.2 mV and 0.2 ng/mL for YF, −2.2 ± 3.6 mV and 0.6 ng/mL for DN, and −4.2 ± 5.2 mV and 2.82 ng/mL for CHK, respectively.

**Figure 8:**
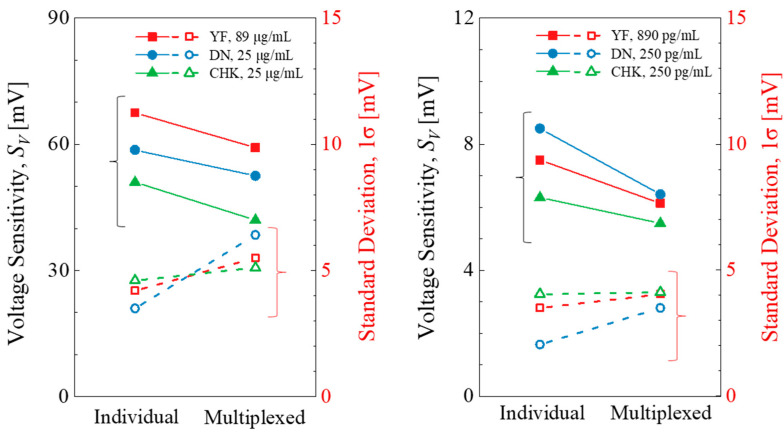
*S_V_* and 1σ variation for the individual and multiplexed detection at the highest (**left**) and lowest (**right**) target concentrations.

Figure 8 compares the *S_V_* and the standard deviation (1σ) for individual and multiplexed responses at the highest and lowest concentrations. The multiplexed detection shows a decrease in *S_V_* and an increase in 1σ compared to individual detection. The average reduction in *S_V_* was approximately 22.7%, 4.3%, and 11.8% for YF, DN, and CHK, respectively. When multiple targets are introduced simultaneously onto a sensor surface, they compete for the limited binding sites, thereby reducing sensitivity [36,37]. Moreover, the uneven diffusion among coexisting targets could lead to an additional fluctuation in the measured signals.

Table 1 summarizes the sensing performance of various biosensors used for arbovirus detection. In the individual-target detection scheme, the Si-based EGT achieved significantly lower LODs than other electrochemical and optical sensors [38,39,40,41,42,43,44]. In the multiplexed detection, the Si-based EGT also outperformed colorimetric sensors, showing tens-fold lower LODs. These results demonstrated the superior sensitivity and multiplexing capability of the Si-based EGT array.

### 3.4. Selectivity Test

**Figure 9:**
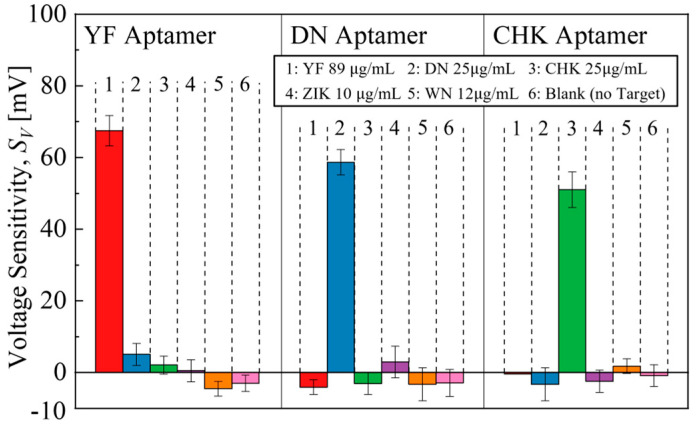
Selectivity tests of aptamer-functionalized EGTs for YF, DN, CHK, ZIK, WN, and blank samples.

To evaluate the specificity of the EGTs, selectivity tests were performed under individual-target conditions, in which each aptamer was exposed to its corresponding target as well as to non-matching targets, as shown in Figure 9. In each case, only the aptamer–specific target pair produced a distinct response, while negligible signals were observed for non-matching targets, even at relatively high concentrations. Blank samples (1 × PBS without targets) used as negative controls also showed a negligible response. Furthermore, the devices were tested with WN and ZIK arboviruses, yielding negligible responses and confirming the retention of high specificity against their respective targets.

## 4. Conclusions

The multiplexed detection of arboviruses using Si-based EGT arrays has been evaluated. The fabricated EGTs showed outstanding intrinsic electrical characteristics, including a *V_TH_* of ~0.8 V, an *SS* of 75 mV/dec, and *I_G_* below 10 pA, which are essential for ensuring uniform signal response in multiplexed detection. Aptamers specific to YF, DN, and CHK were functionalized across a 4 × 4 EGT array at room temperature prior to exposure to individual or multiplexed target solutions. For individual-target detections, the aptamer–target pairs (YF, DN, CHK) showed negative *V_TH_* shifts at varying target concentrations, whereas the non-matching pairs showed negligible *V_TH_* change. The extracted LODs were as low as 38.6 pg/mL for YF, 95.2 pg/mL for DN, and 1.6 ng/mL for CHK, respectively. For multiplexed detections, the *I_D_*–*V_G_* characteristics shifted with increasing target concentration; however, the extracted sensitivities were degraded by 22.7%, 4.3%, and 11.8% for YF, DN, and CHK, respectively, compared to individual detection. The degradation of *S_V_* and 1σ affected the LOD values, which were 0.2 ng/mL for YF, 0.6 ng/mL for DN, and 2.8 ng/mL for CHK. However, these LODs are still significantly lower than those of other methods. The specificity test confirms that only the aptamer–specific target pair produced a distinct response, while negligible signals were observed for non-matching targets. These results demonstrate the promise of Si-based EGT arrays as a scalable, cost-effective diagnostic platform for arbovirus detection and point-of-care testing.

## Figures and Tables

**Table 1:** Performance comparison of various biosensors for arbovirus detection.

Detection Scheme	Biomarker	Method/Device	Dynamic Range	LOD	Ref.
Individual	YFV-NS1	Optical, EIS/Microplate, Au electrode	72 ng/mL–9.2 μg/mL 460 pg/mL–46 μg/mL	39 ng/mL 127 pg/mL	[38] [39]
	DENV-NS1	EIS/SPCE, GCE	1 ng/mL–200 ng/mL 0.92 ng/mL–92 ng/mL	0.3 ng/mL 0.36 ng/mL	[40] [41]
	CHIKV-NS3 CHIKV Antigen	Optical, EIS/POF, PCE	520 pg/mL–10 μg/mL 100 pg/mL–1 μg/mL	520 pg/mL 100 pg/mL	[42] [43]
	YFV-NS1 DENV-NS1 CHIKV-E2	FET/Si EGT	890 pg/mL–89μg/mL 250 pg/mL–25 μg/mL 250 pg/mL–25 μg/mL	38.6 pg/mL 95.2 pg/mL 1.6 ng/mL	This work
Multiplexed	YFV-NS1 DENV-NS1 ZEBOV	Optical/Paper Strip	75 ng/mL–500 ng/mL	150 ng/mL for all	[44]
	YFV-NS1 DENV-NS1 CHIKV-E2	FET/Si EGT	890 pg/mL–89 μg/mL 250 pg/mL–25 μg/mL 250 pg/mL–25 μg/mL	0.2 ng/mL 0.6 ng/mL 2.8 ng/mL	This work

Abbreviations: CHIKV-NS3, chikungunya virus nonstructural protein 3; ZEBOV, Zaire Ebola virus; EIS, electrochemical impedance spectroscopy; SPCE, screen-printed carbon electrode; GCE, glassy carbon electrode; POF, plastic optical fiber; PCE, PVC electrochemical-based analytical device.

## Data Availability

The original contributions presented in this study are included in the article. Further inquiries can be directed to the corresponding author.
